# Validation of Human Skeletal Muscle Tissue Chip Autonomous Platform to Model Age-Related Muscle Wasting in Microgravity

**DOI:** 10.21203/rs.3.rs-2631490/v1

**Published:** 2023-03-29

**Authors:** Maddalena Parafati, Shelby Giza, Tushar Shenoy, Jorge Mojica-Santiago, Meghan Hopf, Legrand Malany, Don Platt, Paul Kuehl, Isabel Moore, Zachary Jacobs, Gentry Barnett, Christine Schmidt, William McLamb, Paul Coen, Twyman Clements, Siobhan Malany

**Affiliations:** University of Florida; University of Florida and Space Tango; University of Florida; University of Florida; AdventHealth; Micro-gRx, INC; Micro Aerospace Solutions, INC; Spae Tango, LLC; Space Tango; Space Tango; Space Tango; university of Florida; Space Tango; Space Tango; Space Tango; University of Florida

**Keywords:** human primary muscle myocytes, microfluidic PDMS chip, autonomous CubeLab^™^, Tissue chips in microgravity, age-related muscle atrophy

## Abstract

Microgravity-induced muscle atrophy experienced by astronauts shares similar physiological changes to muscle wasting experienced by older adults, known as sarcopenia. These shared attributes provide a rationale for investigating microgravity-induced molecular changes in human bioengineered muscle cells that may also mimic the progressive underlying pathophysiology of sarcopenia. Here, we report the results of an experiment that incorporated three-dimensional myobundles derived from muscle biopsies from young and older adults, that were integrated into an autonomous CubeLab^™^, and flown to the International Space Station (ISS) aboard SpaceX CRS-21 in December 2020 as part of the NIH/NASA funded Tissue Chips in Space program. Global transcriptomic RNA-Seq analysis comparing the myobundles in space and on the ground revealed downregulation of shared transcripts related to myoblast proliferation and muscle differentiation for those in space. The analysis also revealed differentially expressed gene pathways related to muscle metabolism unique to myobundles derived from the older cohort exposed to the space environment compared to ground controls. Gene classes related to inflammatory pathways were uniquely modulated in flight samples cultured from the younger cohort compared to ground controls. Our muscle tissue chip platform provides a novel approach to studying the cell autonomous effects of microgravity on muscle cell biology that may not be appreciated on the whole organ or organism level and sets the stage for continued data collection from muscle tissue chip experimentation in microgravity. Thus, we also report on the challenges and opportunities for conducting autonomous tissue-on-chip CubeLab^™^ payloads on the ISS.

## Introduction

The number of National Aeronautics and Space Administration (NASA) sponsored payloads to the International Space Station (ISS) National Lab increased nearly 300% between 2010–2020 according to the Center for the Advancement of Science in Space (CASIS) annual reports. The growth was particularly in the areas of technology development and demonstration and life science research, due to the NASA commercial resupply program, increased financial support for space-based research and availability of implementation companies offering a diversity of custom payload hardware. The Tissue Chips in Space initiative, a cooperation between the National Institutes of Health (NIH) and NASA has leveraged increased access to the ISS with advanced tissue engineering and microfabrication approaches to investigate microgravity-induced changes on various tissue functions including those associated with cardiac dysfunction, decreased bone density, muscle wasting and immunosuppression. The long-term goals of the initiative are to uncover molecular mechanisms associated with disease progression related to aging, to advance lab-on-chip technologies for the evaluation of therapeutics to counteract pathologies in vitro with clinically relevant physiology, and to maintain prolonged tissue cultures to study microgravity and radiation effects on chronic human diseases progression.

**Figure 1:**
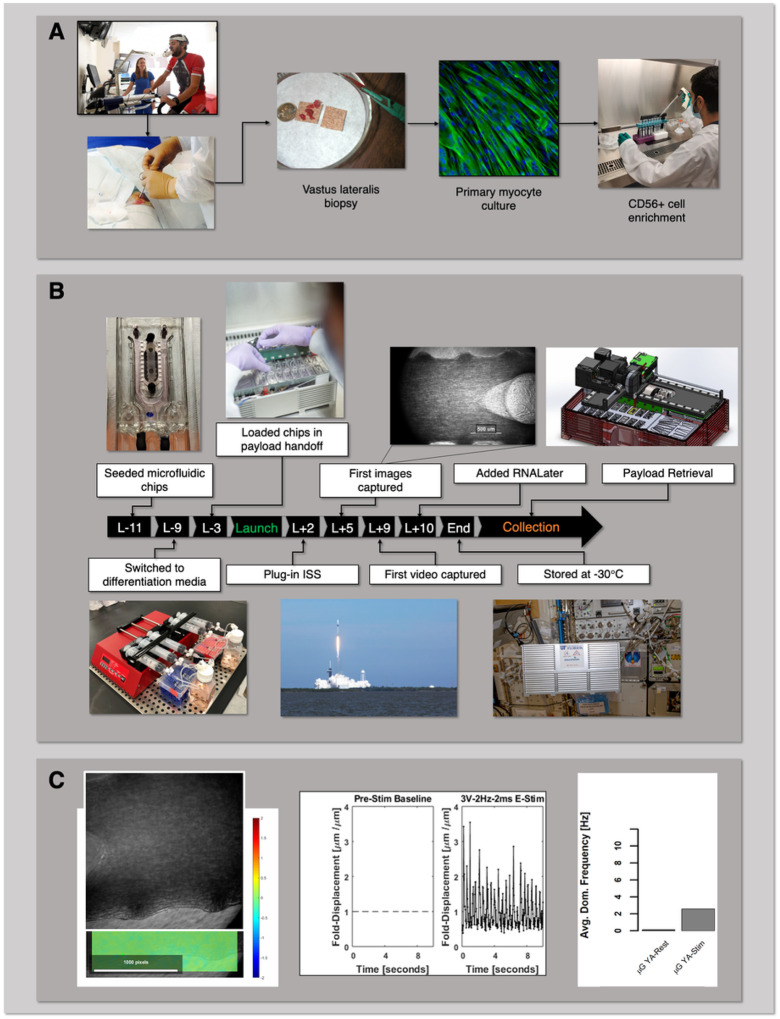
Experimental design, implementation, and automation of the skeletal muscle MPS payload for the International Space Station. a Human cell banks. Skeletal muscle biopsies were obtained from the vastus lateralis from volunteers (AdventHealth, Orlando). Isolated muscle precursor cultures were enriched for CD56+ (myogenic) cells. b Experimental flight timeline. Tissue chips were seeded with myoblasts pre-differentiated, loaded into the CubeLab^™^ and launched to the ISS on SpaceX CRS-21. Crew members installed the PAUL on the EXPRESS rack locker and the on-orbit experiment was initiated after plug-in. 10 days post launch, crew members, moved the payload to cold stowage following experiment termination with RNALater. c On-orbit real-time telemetry. Recording of YA-derived myobundle during electrical stimulation on-orbit and resultingdominant frequency of contraction determined by Fast Fourier Transform of the time series signal of displacement.

Our team at the University of Florida has developed a skeletal muscle microphysiological system that, through multiple flights to the ISS, seeks to correlate changes in global transcriptomics and muscle biomechanics with accelerated muscle dysfunction induced in microgravity^1^. In an increasing aging population, progressive muscle atrophy or sarcopenia leads to a dramatic decline in mobility^2^, and yet remains without effective therapeutic options^3,4^. Astronauts, on the other hand, experience significant muscle loss in an accelerated timescale, 20% over two weeks, compared to 1–2% per year for the typical person over age 35^5,6^, such that complete prevention is not attainable with rigorous exercise^7^. In collaboration with AdventHealth, Translational Research Institute (TRI), we obtained percutaneous biopsies of the vastus lateralis from five young, athletic (<40 yrs, YA) and five older, sedentary (>60 yrs, OS) volunteers, and derived muscle precursor cells that were then purified for CD56^+^ selection, a cell surface marker expressed in myogenic cells (Fig 1A). In this communication, we report on the differences in contractile function and gene expression profiling between the flight YA- vs OS-derived myobundles and ground YA- vs OS-derived myobundles^1^. Results from our previous work incorporating the same cell stocks indicated that the precursor cells retained aspects of the donors’ phenotype to provide distinct engineered muscle myobundles^1^. The muscle myobundles were perfused in microfluidic devices custom-designed for compatibility with flight hardware (Fig. 1B). By sending our muscle tissue chips to the ISS, we tested the hypothesis that microgravity-induced cellular and molecular changes in the muscle cells may mimic salient features of muscle dysfunction that may be differentially pronounced in the myobundles derived from young adults compared to their older counterparts.

In our first flight under the Tissue Chips in Space program, 16 muscle tissue chips were integrated into an autonomous Cubelab^™^, engineered by our NASA-certified implementation partner Space Tango, and launched on SpaceX CRS-21 on December 6, 2020 to the ISS for a 10-day of experiment in microgravity. In this communication, we demonstrate implementation of tissue chips for space research, describe results of transcriptomic studies collected between ground and space treated tissue chips, and share the technical advancements, challenges, and opportunities in deployment of an autonomous human muscle tissue chip payload for modeling musculoskeletal disease in microgravity. Through iterative testing in microgravity, our tissue chip platform informs us of new therapeutic targets and treatments for age-related muscle wasting on Earth.

Our muscle microphysiological system (MPS) includes engineered three-dimensional myobundles perfused in a custom designed polydimethylsiloxane (PDMS) device with or without embedded electrodes, as previously described (Fig. 1B)^1^. We validated a two-stage differentiation protocol that was implemented for the flight experiment. The flight timeline is outlined in Fig 1B. Eleven days prior to launch (L-11), eight PDMS devices were seeded with pooled myoblasts from the young athletic (YA) cohort and an additional eight devices were seeded with pooled myoblasts from old sedentary (OS) cohorts. The tissue chips were switched from growth media to stage I differentiation media two days later (L-9) and subsequently switched to stage II differentiation media and integrated into a 9U CubeLab^™^ three days prior to launch (L-3). The CubeLab^™^ was designed to maintain environmental controls beyond facility ambient conditions and capture analytics of the tissue chips.The CubeLab^™^ contained the following subsystems: thermal manifold, fluidic control system, movable microscope imaging device, cold flask system to maintain media 4–8°C, conduction incubator, control electronics, and flight computer. During launch, the CubeLab^™^ was housed in a powered accent utility locker (PAUL) and once on the ISS, crew members installed the PAUL into an EXPRESS Rack locker, which provided mechanical, electrical, and network payload interfacing. Once installed, a pre-programmed protocol was initiated as described in Materials and Methods and the module performed autonomously and reported data daily including flow rate, environmental conditions, and images downlinked to a portal via a universal asynchronous transmitter interface.

Images of a pre-defined region of interest in each myobundle were captured using the PDMS micro post as a reference point. Cell image acquisition continued every 12 h and telemetry was downlinked daily to the portal system^8^. We did experience heat buildup from the microscope that limited some fine tuning of images. The cold flask average temperature was 9–10°C throughout the experiment. Telemetry from the CubeLab^™^ recorded an average 6.5% CO_2_, 40–45% humidity, and manifold temperature of 37–42°C. Although the flow rate was set to 125 ml/min, the flow sensor showed off-nominal flow readings between 0–500 ml/min throughout the experiment, possibly due to an air bubble in the flow sensor. The platinum electrodes embedded at the PDMS-media channel interface were wired to the Space Tango circuit board. Thirty minutes of electrical stimulation of 3V, 2Hz, 2msec pulse was applied sequentially to four YA and four OS tissue chips. Pixel displacement was captured from a single chip exhibiting contraction (Fig. 1C). Fold-displacement prior to and during electrical stimulation was analyzed by digital image correlation and was determined to be between 0.5 – 3.0-fold during ten seconds of recording compared to no detectable displacement prior to applying stimulation (Pre-Stim baseline, Fig.1 C). The average dominate frequency was 2.0 Hz. The experiment was planned to continue over 14 days post launch; however, due to heat spikes detected during imaging and the possibility of air blocking the fluid flow, we terminated the experiment early to ensure robust results. The RNALater protocol was manually initiated on day 10 post-launch and each sample was flushed with at least 0.6 ml of reagent to preserve transcriptomic material. The CubeLab^™^ was moved to −32°C cold stowage until payload return.

The payload returned to earth onboard a SpaceX Dragon capsule which splashed down on January 13, 2021. The payload was returned frozen to Kennedy Space Center (KSC) Space Station Processing Facility (SSPF), where the team removed tissue chips and fluid bags from the CubeLab^™^. Inspection of the waste bags indicated that 10 ml of media and 6 ml of RNALater flowed through the tissue chips. Although perfusion of the tissue chips over 10 days in microgravity was less than expected, the samples were sufficiently preserved with RNALater at the termination of the experiment. The RNA quantity and quality for each of the 16 tissue chips was determined by electrophoretic separation on a bioanalyzer and the results are listed in Supplemental Table 1. The flight samples yielded an average of 1.6 ± 0.6 mg of total RNA with average RNA integrity number (RIN) of 7.8 ± 1.0, indicating high RNA integrity and sufficient material for RNA-Seq analysis (RIN values range from 10 (intact) to 1 (totally degraded)). Post launch, we seeded cells from the same CD56+ population into an equivalent lot of PDMS chips and recapitulated the timeline shown in Fig. 1 under similar conditions in a cell incubator as a ground control. The samples were terminated with RNALater and frozen to serve as ground controls to the flight samples. The ground samples provided an average of 1.5 ± 0.3 mg of total RNA with an average RNA integrity number (RIN) of 9.2 ± 1.0. We chose to perform the ground study under standard conditions since we experienced anomalies with the flow sensor in the CubeLab^™^ that are challenging to fully recapitulate. We replicated a similar experiment on a recent flight and analysis are underway directly comparing samples in the same CubeLab^™^ sent to the ISS or incubated on ground. Plans entail comparing results from both flights. Communicating our preliminary results for this first-in-class muscle MPS payload is critical in standardizing and advancing cell-based autonomous systems in low Earth orbit.

**Figure 2:**
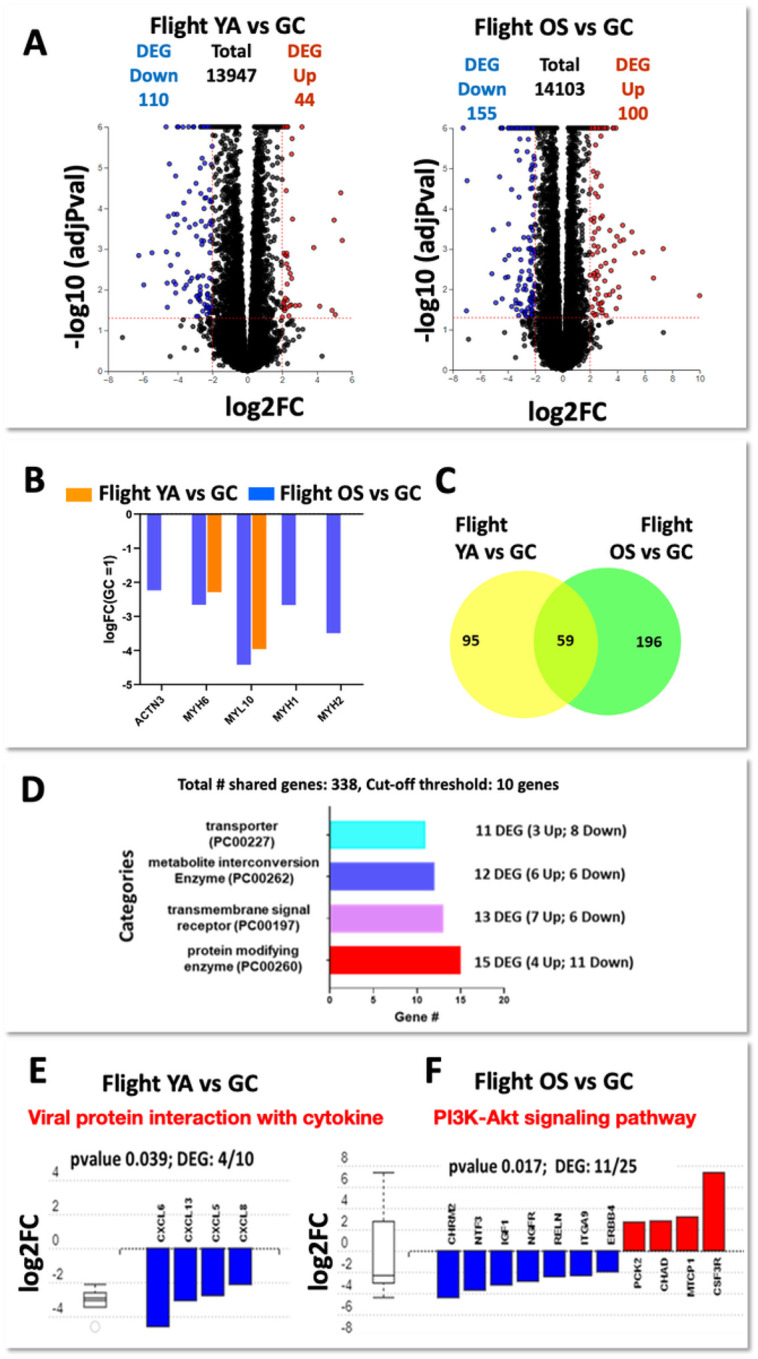
Transcriptional responses of muscle myobundles during flight and ground culture. a, Volcano plots. Differential expressed genes between flight and ground controls for YA (left panel) or OS (right panel). Red: significantly up-regulated genes in flight. Blue: significantly down-regulated genes. Significance determined according to log2 fold change with threshold set at ±2 and -log10 p-value ≤0.05. b. Comparative analysis of differential gene expression changes between flight and ground controls of genes involved in muscle myogenesis and contraction YA (orange bars) and OS (blue bars). c. Venn diagrams. Overlapping and distinct DEGs among Flight YA vs GC and Flight OS vs GC datasets. d. Panther classification system of the 59 shared genes shown in the Venn diagram. The horizontal bar chart identifies the four protein classes and relative number of DEGs of each functional class by applying a cut-off threshold of 10 genes. e. f. Significantly enriched KEGG pathways in response to flight either in YA- or OS-derived myobundles relative to their respective ground controls.

We performed transcriptomic profiling to establish differential patterns of gene expression between muscle tissue chips exposed to microgravity for 10 days compared to tissue chips cultured on ground. Applying a RIN number cutoff of 7.0, the following treatment groups (Labeled in Supplemental Table S1) were performed in triplicate in the RNA-Seq analysis on triplicate chips from the young, athletic group in microgravity (YA MG), the young, athletic ground control (YA GC), the old, sedentary group in microgravity (OS MG), and the old, sedentary ground control (OS GC). The chips analyzed are labeled in the Supplemental Table 1. RNA-Seq results displayed as Volcano plots demonstrated that there was a total of 154 and 255 differential expressed genes (DEGs) in YA- and OS-derived myobundles in microgravity compared to their control groups, respectively (2-fold change, false discovery rate (FDR) < 0.05, Fig. 2A). In the data set, 110 and 44 DEGS were down- and up-regulated in YA-derived tissue chips, respectively, in microgravity compared to ground controls; the genes are listed in Supplemental Table 2. In addition, 155 and 100 DEGs were down- and up-regulated in OS-derived tissue chips, respectively, in microgravity compared to ground controls; these genes are listed in Supplemental Table 3. There was no significant difference in the DEGs between myobundles receiving 30 min of electrical stimulation prior to RNALater addition and those not receiving electrical stimulation. Therefore, we chose to focus bioinformatic analysis on non-electrically stimulated flight and ground samples that will serve as a baseline model for comparison to future flights that will include a repeat set of non-electrically stimulated tissue chips and a set of tissue chips electrically stimulated for 30 min every 12 h for eight days.

Previously, we reported that genes related to skeletal muscle myogenesis and myopathy are similarly upregulated in YA- and OS-derived myobundles differentiated for two weeks compared to their respective myoblast stages indicating transition to mature myotubes^1^. As such, we implemented the same differentiation protocol for the current experiments. Our flight results suggest tissue chips exposed to the microgravity environment may induce specific shifts in the expression of myosin heavy- and/or light-chain genes. We observed that myogenic genes a-actinin-3 (ACTN3), myosin heavy chain 1, 2, and 6 (MYH1, MYH2, MYH6), and myosin light-chain isoform 10 (MYL10) were significantly downregulated in OS-derived myobundles exposed to microgravity compared to ground controls (Fig. 2B). Of these genes, MYH6 and MYL10 were also downregulated in YA-derived microgravity versus ground controls. Studies conducted on astronauts have shown that skeletal muscles remodel their metabolic profile and transition fiber types to allow for metabolic adaptation to the spaceflight environment^9–11^. In humans, type 1 fibers are oxidative, and type 2A and 2X fibers primarily rely upon glycolytic metabolism^12^. Microgravity stimulates slow-to-fast fiber transition^13,14^, whereas age and exercise induce fast-to-slow fiber transition^15,16^. In our in vitro model, these alterations in myosin gene expression composition are pronounced in the OS tissue chips, indicating a down-regulation of mRNA levels of MYH1 and MYH2 by 2.6 and 3.5-fold. These genes encode for Myosin heavy chain-1 (fast type IIX muscle fibers) and Myosin heavy chain-2 (fast type IIA muscle fibers), respectively. In addition, the fast type II fiber-related gene, ACTN3 is downregulation by 2.2-fold (Fig. 2B). Both YA- and OS-derived myobundles showed down-regulation of the MYH6 gene, present in slow-twitch type I fibers, by 2.3- and 2.7-fold respectively. The downregulation of MYL10, which enables calcium binding in the motor protein, in both YA- and OS-derived myobundles supports a shift in fiber type. Overall, these results suggest that the microgravity environment my affect the fiber type transition to type I oxidative muscle fibers predominately in the myobundles derived from the aged cohort. This may be confirmed in the follow-on flight opportunity.

Applying the Panther classification system, we observed 59 shared genes between the YA- and OS-derived tissue chips in microgravity compared to ground controls (Fig. 2C)^17^. The analysis identified four strongly impacted protein categories (log2 FC ≥ ±2) including protein modifying enzyme class, metabolite interconversion enzyme class, transmembrane signal receptor class, and transporter protein class with 15, 13, 12 and 11 significantly modulated genes, respectively (Fig. 2D). The values for these gene targets are displayed as heatmaps in Supplemental Fig. 1A–D.

Skeletal muscle is characterized by elevated utilization of glucose and high fatty acid oxidation rates^18^; our data suggest that genes encoding for enzymes involved in the regulation of glucose are dysregulated in flight samples, regardless of the young or old phenotype. These results suggest metabolism remodeling. In the protein modifying enzyme class, we observed the downregulation of pyruvate dehydrogenase kinase 4 (PDK4), a key metabolic enzyme that regulates muscular pyruvate metabolism by inhibiting pyruvate dehydrogenase (PDH) by 3.3- and 2.8-fold in YA- and OS-derived tissue chips, respectively, when exposed to microgravity compared to ground controls (Supplemental Fig. 1A). PDK4 mRNA are markedly increased in human skeletal muscle during prolonged exercise^19^. The flight samples exhibit decreased PDK4 mRNA in the microtissues, subsequently inducing entry of carbohydrate-derived pyruvate into the Krebs cycle and undergoing oxidative phosphorylation with ATP production. Furthermore, YA and OS flight microtissues were characterized by increased mRNA levels of genes encoding for key rate-limiting enzymes involved in glycolysis as shown in the metabolite interconversion enzyme protein class (Supplemental Fig. 1B). Phosphofructokinase (PFKP), a key enzyme in glycolysis, was up-regulated by 2.5 and 2.0-fold in in YA- and OS-derived tissue chips, respectively, in microgravity compared to ground controls. Phosphoenolpyruvate carboxykinase 2 (PCK-2) gene, which encodes for an enzyme involved in glucose biosynthesis, was also up-regulated by 2.0 and 2.7-fold in YA and OS flight samples, respectively, compared to ground controls. These results indicate the potential response of the flight samples to increased glucose metabolism. Other enzyme-encoding genes in the metabolite interconversion enzyme protein class similarly up-regulated in both tissue chip sets in microgravity compared to ground controls included nitric oxide synthase (NOS1) and arginase 2 (ARG2) genes, two enzymes that metabolize L-arginine (Supplemental Fig. 1B). Evidence indicates that L-arginine protects muscle cells from wasting in vitro in an mTORC1-dependent manner^20,21^ by promoting protein anabolism in myocytes through the involvement of the nitric oxide (NO) and mTOR/p70S6K signaling pathways^22,23^. Also, NO has been associated with skeletal muscle-wasting diseases, sarcopenia, and cachexia^24^ and activation of NO during muscle injury is critical in the early phases of the skeletal muscle repair processes^22,25^. However, the role of NO in myocyte protein synthesis under microgravity conditions needs to be elucidated.

One of the top 5 significantly upregulated genes in both YA (5-fold) and OS (7.3-fold) flight samples compared to ground controls encodes granulocyte colony-stimulating factor receptor 3 (CSF3R) in the transmembrane receptor class (Supplemental Fig 1C, Supplemental Tables 2 and 3). CSFR is expressed in skeletal muscle during development and in adults^26,27^. The receptor plays pivotal roles in skeletal myocyte development, regulation of muscle regeneration and is increased in injured skeletal muscle. Treatment with CSF has been shown to enhance muscle regeneration processes by promoting the proliferation of satellite cells^27^. It is not clear if high CSF3R levels may dysregulate muscle regeneration pathways in humans. Our data suggest a potential modulation of the CSF / CSFR axis in the regulation of muscle volume in the tissue chips exposed to microgravity. On the other end of the spectrum, members of the transporter class including from the potassium and sodium voltage gated channel families are significantly downregulated in flight samples compared to ground controls (Supplemental Fig. 1D). Members of these ion channel genes are expressed in the plasmalemma, T tubules, and sarcoplasmic reticulum of skeletal muscle responsible for controlling muscle fiber electrical properties and signal transduction of the action potential^28^. The potassium channel subfamily A member 10 (KCNA10) channel is downregulated in both YA (2.3-fold) and OS (4.6-fold) flight samples compared to ground controls and the sodium channel alpha subunit 3 (SCN3A) channel is downregulated in YA (2.6-fold) and OS (3.8-fold) flight samples compared to ground controls (Supplemental Tables 2–3). The alpha subunits 2 and 7 are also downregulated in OS-derived flight samples by 2.4- and 2.5-fold, respectively, indicating the downregulation of muscle fiber activity may be more pronounced in the aged-derived myobundles. The set of tissue chips we are investigating are not undergoing electrical stimulation. In subsequent flights, we have applied electrical stimulation to half of the flight samples and the resulting data may be compared to results from our first flight to understand how microgravity affects the electrical properties of the myobundles.

Functional enrichment using Kyoto Encyclopedia of Genes and Genomes (KEGG) pathway analysis in YA-derived myobundles revealed enrichment of downregulated CXC chemokine signaling genes (DEGs=4/10; P = 0.039, Fig. 2E). In healthy skeletal muscle, the chemokine expression profile is low, however, many chemokines are induced or upregulated in dystrophic muscle^29–31^. In addition, we observed only in YA-derived flight samples compared to ground controls, downregulation of JAK-STAT signaling including decreased expression levels of 1) transcription factor Jun B proto-oncogen (JUNB), 2) the suppressor of cytokine signaling 1 (SOCS1) and 3) the cytokine inducible SH2 containing protein (CISH) by 2.13-, 2.8- and 4.6-fold, respectively (Supplemental Table 2). Since our differentiated muscle myobundles do not contain a satellite cell niche, the downregulation of the chemokine signaling, and JAK/STAT pathway indicates a compensatory mechanism to protect against muscle wasting. JunB has been suggested to play a role in both the maintenance and hypertrophy of skeletal muscle mass and to stimulate protein synthesis independently of Akt and mTOR^32^. Several studies have suggested that the activity of JunB is finely regulated in skeletal muscle. For instance, the expression of JunB (AP-transcription factor subunit), can be induced by exercise^33^. Our findings are consistent with the observations that JunB expression is decreased during various types of atrophy^34,35^.

In OS-derived myobundles exposed to microgravity, we did not observe modulation of the CXC chemokine pathways. Uniquely, the KEGG pathway analysis revealed that DEGs were enriched in the phosphoinositide 3-kinase (PI3K)/threonine protein kinase B (AKT) signaling pathway (DEGs=11/25; P = 0.017, Fig. 2F). The PI3K/AKT pathway is a predominant pathway controlling skeletal muscle metabolism. Exogenous insulin-like growth factor-1 (IGF-1) targeting PI3K/AKT is known to increase skeletal muscle protein synthesis via activation of Akt phosphorylation and mTOR activation and has emerged as a potential target for mitigating skeletal muscle loss due to microgravity^36^. IGF-1 expression was downregulated by 3.2-fold in OS flight samples compared to ground controls (Fig. 2F, Supplemental Table 3). The dysregulation of IGF-1 downstream signaling pathways and the reduction of myosin content in OS flight samples suggest a negative impact on muscle microtissue health specific to muscle cells from older adult-derived myobundles.

Next, we evaluated gene enrichment and functional annotation of DEGs using the Database for Annotation, Visualization, and Integrated Discovery (DAVID) bioinformatic tool. The relevant Gene Ontology (GO) annotations were determined by filtering each contrast with p-values ≤ 0.05, logFC ≥ ±2 and DEG per group ≥ 14. Functional annotation of proteins encoded by DEGs with increased or decreased enrichment compared with their controls were classified according to their associated biological processes, molecular functions, and cellular components. Relevant GO ontological groups were visualized using bubble plots presented in Supplemental Fig. 2A, B and a summary of the detailed results is listed in Supplemental Tables 4 and 5 for YA flight versus ground and OS flight versus ground, respectively. Analysis of cellular component in YA and OS revealed the most significant terms are GO:0016021~integral component of membrane and “GO:0005886~ plasma membrane”, respectively. Regarding the integral component of membrane in YA, a total of 18 DEGs were up- and 33 were down-regulated (Supplemental Table 4). In the OS, a total of 38 were up- and 55 were down-regulated, respectively within the plasma membrane term (Supplemental Table 5). At the level of molecular function, the GO analysis showed the most significant terms in YA and OS are GO:0042802~ identical protein binding and GO:0005509~ calcium ion binding with 5 up- and 13 down- and 7 up- and 18 down-regulated genes, respectively. Finally, biological process analysis, DEGs are mainly enriched in GO:0007165~ signal transduction with 10 up- and 13 down-regulated genes.

Human pathophysiological adaptations to the microgravity environment induce skeletal muscle disorders, including loss of muscle mass, decreased muscle force, fiber-type shift, and metabolic alterations that put astronauts at risk for injury during spaceflight operation and are of great concern for long-term space missions^37^. While molecular mechanisms triggering skeletal muscle remodeling and dysfunction remain to be fully elucidated, the findings from studying humans, rodents, and in vitro systems over the years in simulated and real microgravity has highlighted the various factors, both direct and systemic, that contribute to spaceflight induced muscle atrophy and have highlighted mechanisms, particularly catabolic effects, underlying microgravity-induced muscle tissue loss^38,39^. Several questions remain with respect to how these underlying mechanisms contributing to muscle loss in space mimic the salient features of age-related muscle dysfunction. For example, what are the molecular events controlling metabolic adaptations of muscle cells to microgravity and how are the expression levels of biomarkers and mechanisms modulated in microgravity to regulate skeletal muscle differentiation and biomechanics? As more cell and tissue-based experimentation is performed in the microgravity environment, we can appreciate the impact of microgravity at the tissue level to reveal underlying mechanisms contributing to accelerated decline in muscle properties of clinical relevance. These in vitro disease models may be used as space-based platforms to develop countermeasures and biomarkers to preserve skeletal muscle health particularly in the aging population, diagnose the disease early, and ameliorate the healthcare burden associated with sarcopenia. Thus, an aging disease model that recapitulates age-related muscle dysfunction accelerated by microgravity is essential for functional analysis and has the advantage of excluding the effects of systemic factors and allowing for evaluation of molecular mechanisms at the cellular level.

From results of our first flight, under the Tissue Chips in Space program, we have demonstrated tissue-on-chip experimentation on the ISS in an autonomous laboratory and advanced on-orbit tissue chip imaging, as well as provided proof of concept for collecting real-time muscle contraction telemetry. Overall, we demonstrate that our engineered 3D skeletal muscle MPS has promise to model skeletal muscle dysfunction and reveal corresponding molecular pathways modulated in microgravity that may mimic manifestation of age-related muscle atrophy. Collectively, our data demonstrated that we could identify genes belonging to protein modifying enzyme, transmembrane signal receptor, metabolite interconversion enzyme, and transporter gene classes that are modulated in tissue chips differentiated in microgravity compared to ground controls. The downregulation of key myogenic genes, MYH1, MYH2, MYH6, MYL10, ACTN3, particularly in the OS-derived flight samples compared to ground controls, suggest impaired skeletal muscle differentiation and sensitization of the OS-derived flight samples. The significant upregulation of CSF3R in both cohorts in microgravity suggests inflammatory pathway induced proliferation of regenerating myoblasts is activated. In YA flight samples, cytokine signaling is suppressed as seen by the KEEG pathway analysis and the downregulation of CXC chemokine receptors. In the OS flight samples, the PI3K/AKT pathway is modulated, suggesting activation of pathways controlling muscle metabolism and protein synthesis.

Our team learned valuable information regarding subsystem performance that we have implemented on Space-X CRS 25 (unpublished data) to advance the lab-on-chip model and functionality and provide comparative omics analysis. Data collected from SpaceX CRS-25 will include repeat experimentation of tissue chips derived from the same lot of YA- and OS-pooled cells and non-electrically stimulated over the same time course shown in Fig. 1B. An additional set of differentiated tissue chips are electrically stimulated every 12 h for eight days once installed on the ISS. Future experiments on SpaceX CRS-26 include analyzing electrically stimulated tissue chips in the absence or presence of an anti-atrophic natural product.

### Challenges and Opportunities

Tissue chip platforms are disruptive technologies on Earth to advance insights into human biology early in the drug discovery process. These microphysiological systems hold promise to translate discoveries faster to the clinic and increase efficiency, effectiveness, and decrease costs. This is particularly critical where animal models do not recapitulate human disease pathology and toxicity. However, these technologies still need further development for successful adoption and FDA acceptance. Translation of terrestrial tissue chip platforms to in-space versions and validating the technological approach is immensely challenging and a longer-term commitment. In our first experiment, we were successful in operating the CubeLab^™^ autonomously over several days and capturing images of the tissue chips. We maintained CO_2_ levels and sterility in the CubeLab^™^ environment. We encountered challenges in controlling heating, cooling, and humidity in the closed environment to mimic a cell incubator. High humidity levels contribute to corrosion of electronics, but too low humidity levels contributed to evaporation in the microfluidic devices and introduction of air bubbles which interfered with the flow sensor readings. We also determined that the microscope computer processor needed to be mounted more securely to the top of the CubeLab^™^ to ensure proper heat transfer to the external structure of the CubeLab^™^. The team decided to terminate the experiment earlier than planned and successfully initiated the RNALater step remotely, which we believe preserved the biology and allowed us to collect and analyze the data communicated here. Despite the hurdles in integrating muscle microtissues into a miniaturized, autonomous laboratory, the ability to modify the experimental protocol remotely to maximize scientific performance and preserve biology is a major advantage. The other major advantage is that the experiment did not take crew time to complete. A unique aspect of our CubeLab^™^ experiment is that it housed microtissues derived from phenotyped donors and we can compare effects on gene expression in myobundles exposed to microgravity with genetically matched ground control samples, setting the stage for future precision medicine experiments to be conducted in the space environment. The novelty of our experimentation in space is the integration of tissue chips into an autonomous laboratory and the ability to control experimental conditions and capture real-time analytics that advance our capabilities to perform non-invasive, long duration experiments to collect normative data sets between flight and ground. Our scientific study helps push the boundaries of tissue engineering conducted on the ISS to study age-related alterations at the cellular and molecular level in microgravity. Thus, communicating results of these first experiments is critical for standardization and adaption of these technologies for space use. In addition, iterative testing in space and acknowledging lessons learned will increase robustness, affordability, and accessibility of conducting tissue chip platforms in lower Earth orbit. Eventually, biomedical science payloads need to be conducted autonomously for in-space operation with minimum-as-possible interaction by the crew away from the ground control center. Preliminary flight experiments point toward the utility of space-based MPS platforms in contributing towards more accurate disease models, as the space environment is a novel stressor – it results in data and insights otherwise not obtainable on Earth. To advance the understanding of the value microgravity can add will require more data through iterative, focused, and concerted efforts to reach the goal of providing insights to disease progression for age-related chronic diseases and uncover novel therapeutics and to counteract these health conditions on Earth.

## Materials And Methods

### Myoblast Isolation

Vastus lateralis biopsies were obtained from volunteers at the Translational Research Institute at AdventHealth, Orlando as previously described (Giza et al IRBNET #554559). Satellite cells from individual donors were isolated, pre-plated to remove fibroblasts and pooled by donor cohort, including five young active (YA; 21–40 years) and five older sedentary (OS, 65–80 years) prior to cryopreservation and shipment to the University of Florida through a Material Transfer Agreement and in the absence of any patient identifiable information. Muscle myoblasts were thawed and then cultured on T75 flasks coated with collagen I in Skeletal Muscle Growth medium (PromoCell, Heidelberg, Germany) to confluence. Immunopurification of mononuclear myoblasts was performed using mouse monoclonal 5.1 H11 anti-CD56 antibody (DSHB Hybridoma Bank, Iowa City, IA). CD56+ myoblasts were confirmed by FACS as described, (Giza et al.).

### Preparation of Bioengineered Myobundles Pre-flight

Custom microfluidic chips made from polydimethylsiloxane (PDMS) and containing two platinum 22-gauge electrode leads were obtained from Micro-gRx, INC, (Orlando, FL), sterilized in ethanol and autoclaved at 150°C for 30 min prior to use. Cell seeding was performed at NASA’s Kennedy Space Station Processing Facility (SSPF). Injectable hydrogel mixtures (3.3 mg/mL rat tail collagen I, and 22% (v/v) Matrigel) were combined with YA- and OS-derived CD56+ enriched myoblasts. The cell-laden hydrogels were injected into the PDMS chips to a final cell density of 15 and 20 million cells/mL for YA-and OS-derived cells, respectively. Cells were allowed to polymerize at 37°C for 60 min and seeding ports were sealed using polylactic acid (PLA, Makerbot, Brooklyn, NY) plugs. Chips were perfused with degassed Skeletal Muscle Growth media supplemented with 0.1 mg/ml Primocin (InvivoGen, San Diego, CA) at a constant flow rate of 1 mL/h at 37°C and 5% CO_2_. After 48 h, the culturing medium was changed to degassed differentiation media (MEM-α, 0.5% (v/v) ITS, 2%(v/v) B27,10 μM DAPT, 1 μM Dabrafenib, 20 mM HEPES, pH 7.3 and, 0.1 mg/mL Primocin) and delivered at an intermittent rate of 125 mL/min for 1 min every 8 h for 6 days.

### Tissue Chip Payload Assembly

The sterile CubeLab^™^ hardware was flushed with sterile Phosphate-Buffered Saline (PBS) followed by priming with degassed flight media II (MEM-α, 0.5% (v/v) ITS, 2%(v/v) B27, 20 mM HEPES, pH 7.3 and, 0.1 mg/mL Primocin). Effluent was collected, plated, and incubated at 37°C for 5 days (control samples as well). No colonies were present. 48 h prior to launch (L-2), tissue chips were exchanged with 1 ml media, disconnected from the syringe pump, inspected under a 10x microscope and 16 chips (8 YA and 8 OS) were integrated into a custom designed 9U CubeLab^™^ (Space Tango, Lexington KY). Flight chips were secured PDMS-side down onto fluid connection ports which allowed for fluid-tight mating to the manifold chip posts via press fit connections. A low pulsatile annular gear pump and Takasago-modified valves, controlled fluid to each chip. Two thermoelectric coolers located on the opposite side of the chips ensured consistent temperature (37–39°C). The stimulation circuit was connected to eight chip’s stimulation leads via blue wire and connectors custom to the stimulation board mounted on one side of the manifold. 200 ml of chilled, degassed flight media II was added to the source bag and placed into the cold flask (4–8°C). Chilled RNALater (20 mL) was added to a second source bag and placed into the cold flask (4–8°C). Fluid from each chip (200 mL) was routed to general waste. The payload was sealed, purged with 5% CO_2_ using an air gas canister and the thermal protocol was initiated to bring the manifold to 37°C. Humidity was recorded to be 40–45%. Prior to handover, a leak test was performed to verify pressure integrity and the CubeLab^™^ was integrated into the powered accent utility locker (PAUL). Once in the PAUL, the camera system was initiated and calibrated for each chip position prior to handover to NASA at L-1.

### On-orbit Payload Operation

After docking, the CubeLab^™^ was installed by crew members and operated from within the PAUL. After install, the following protocols were initiated: 1) The *Media Exchange-Waste* event. During this event, media was flowed to each chip every 8 h at 125 mL/min for 1 min and output volume was sent to general waste. 2) The *Z-Stack* event. During this event, the microscope (10× magnification) was moved to chip *N* and captured six phase contrast slices at 12 mm increments at each chip’s pre-defined position immediately before and after each Media-Exchange-Waste event. 3) The *Stimulation & Video* event. During this event, the microscope was moved to chip Ns ideal position and video was recorded at 30 fps for 40 s (pre-stimulation phase). At the 41 s mark after video initiation, a stimulation waveform on chip *N* was elicited and video was recorded for an additional 40 s (stimulation phase). At the 82 s mark after video initiation, the stimulation waveform ceased, and video was recorded for 40 s (recovery phase) for total of 120 s of video. 4) The *Fixation* event. During this event, RNALater was initiated to each chip at 125 ml/min for 5 min at termination of the experiment. Crew members removed the CubeLab^™^ from the PAUL and transferred to the ICEBERG cold stowage asset, at −35°C. Continuous telemetry from the CubeLab^™^ environment was recorded in the customer dashboard portal. Z-stacked images of each chip per day and the stimulation video were downlinked to the portal system. An open-source digital image correlation (DIC) algorithm based in MATLAB (Mathworks, Natick, MA) was used to quantify displacements at the region of interest in the tissue chip as previously described (Giza et al.).

### Post-flight RNA Extraction

Payload splashdown from SpaceX CRS-21 occurred on January 14, 2021, and the CubeLab^™^ was transferred from a Double Cold Bag (conditioned at −32°C) to a −80°C freezer at NASA’s Kennedy SSPF processing laboratory where the CubeLab^™^ was de-integrated and chips were removed and immediately processed for RNA isolation. Flight chips in RNALater were lysed in RLT buffer (Qiagen, Germantown, MD) prepared with B-mercaptoethanol. RNA isolation was then performed using RNeasy Plus Mini Kit according to manufacturer’s instructions. The integrity of the extracted RNA was assessed by Agilent 2200 Tapestation system (Agilent, Santa Clara, CA),

### RNA Sequencing Analysis

Libraries of extracted RNA samples were built using the NEBNext^®^ Ultra^™^ Directional RNA Library Prep Kit for Illumina (NEB, USA) RNA sample preparation kit and yielded fragments with 220–700 base pairs. The qualified fragments were ligated with 60 adapters, amplified, and submitted for sequencing by Illumina NovaSeq 6000 (Illumina, San Diego, CA) to generate paired end reads with a length of 150 bases. The input sequences were trimmed using trimmomatic. Quality control was performed before and after trimming using FastQC (v 0.11.4) and MultiQC^40^ and a total of 50 million reads were generated for each sample yielding coverage in the range of 118 to 220 bases for each sample, Then the input sequences were aligned to the transcriptome using the STAR aligner, version 2.7.9a^41^. Transcript abundance was quantified using RSEM (RSEM v1.3.1)^42^, and genes with insufficient average counts were excluded from further statistical analysis. Differential expression analysis was performed using the DESeq2 package^43^, with an FDR-corrected P-value threshold of 0.05. The results were further filtered to extract transcripts showing a 2.0-fold change (log2FC) in either direction.

### Differential Expression and Functional Annotation Analysis of RNA-Seq Data

Differentially expressed genes (DEGs) from YA- and OS-MG samples were normalized to the matching GC group and reported as log2 of the fold change (log2FC). Fold induction values were averaged for all experiments performed as experimental triplicates for each cohort. RNA-seq data were analyzed by iPathwayGuide (Advaita Bioinformatics: http://www.advaitabio.com/ipathwayguide.html) by identifying significantly impacted signaling pathways. Volcano plots, which rely on double filtering criterion and display unstandardized signal log2FC against noise-adjusted/standardized signal FDR-corrected P-value, were used to display up- and down-regulated DEGs. Gene Ontology (GO) and Pathway enrichment analysis were performed by comparing DEGs with Kyoto Encyclopedia of Genes and Genomes (KEGG) databases where p < 0.5 was statistically significant.

### Statistical Analysis

Quantification methods are described in Methods Section. Specific tests used are indicated in the Figure legends. P values for significant differences are indicated in the graphs. All graphs show the individual data points used in the analysis.

## Data Availability

All data generated during this study are either included in the manuscript and its Supplementary files or are available from the corresponding authors upon reasonable request. Patient biopsy cells were obtained from AdventHealth Orlando through a Material Transfer Agreement to the University of Florida with restrictions for sharing with a third party. RNASeq data are available at the Gene Expression Omnibus (GEO) database (XXX) and MPS database (XXX).
